# *Withania somnifera* Root Extract Ameliorates PTU‐Induced Hypothyroidism by Regulating Hormone Levels and Gene Expression in Rats

**DOI:** 10.1002/fsn3.71732

**Published:** 2026-04-17

**Authors:** Md. Saud Hossain, Md. Shah Poran Shuvo, M. Maruf Hasan Talukder, Shahidullah, Sumaia Jannat, S. M. Riaduzzaman Niaz, Md Sohel, Md. Khairul Islam

**Affiliations:** ^1^ Department of Biochemistry and Molecular Biology Mawlana Bhashani Science and Technology University Tangail Bangladesh

**Keywords:** autoimmune thyroiditis, D2, gene expression, histopathology, molecular docking, phytochemicals, thyroid hormones, TSHR

## Abstract

Hypothyroidism is a prevalent endocrine disorder characterized by the inadequate production of thyroid hormones (T3 and T4), leading to various physiological dysfunctions. Current treatments often involve synthetic hormone replacement, which is limited by some adverse effects. This study investigated the therapeutic efficacy of a methanolic extract of *Withania somnifera* root (MEWS) in propylthiouracil (PTU)‐induced hypothyroid rats. Forty rats were divided into five groups (*n* = 8): (1) negative control (NC), receiving no treatment; (2) positive control (PC), administered 0.05% w/v PTU to induce hypothyroidism; (3) *W. somnifera* control (WSC), treated with 500 mg/kg/day MEWS alone; (4) *W. somnifera* treatment (WST‐500), receiving 500 mg/kg/day MEWS alongside PTU; and (5) combination therapy (CT‐500), treated with both 500 mg/kg/day MEWS and 0.05% w/v PTU. Serum hormones (TSH, T3, T4) were measured by ELISA, thyroid histopathology was analyzed, and thyroperoxidase (TPO) and thyroglobulin (TG) gene expression were quantified by qPCR. Molecular docking and ADMET profiling were employed to identify bioactive phytochemicals that target the thyroid‐stimulating hormone receptor (TSHR) and Type 2 iodothyronine deiodinase (D2). PTU induction significantly increased thyroid weight (175.8%), TSH levels (4.45‐fold), and TPO/TG expression (4.28 and 3.10‐fold), while reducing T3 and T4 (82% and 75%; all *p* < 0.05). MEWS treatment (WST‐500) significantly reversed these effects, reducing thyroid weight (55.8%), TSH (74.2%), and TPO/TG expression, while restoring T3 and T4 levels to near‐normal (*p* < 0.05). Histopathology revealed reduced fibrosis and improved follicular architecture. A computational study identified phytochemicals with strong binding affinities for TSHR and D2 and favorable ADMET properties. These findings suggest that MEWS may ameliorate hypothyroidism by regulating hormone levels, gene expression, and thyroid morphology, indicating its potential as an adjunct or alternative to conventional hormone replacement therapies for hypothyroidism.

## Graphical abstract

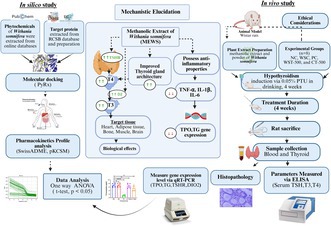

## 1 Introduction

Hypothyroidism is a common endocrine disorder with a global prevalence of 1%–5%, characterized by inadequate thyroid hormone production (low T3/T4) and elevated TSH (Bianco 2024; Zamwar and Muneshwar 2023). In Bangladesh, prevalence ranges from 3.46% to 7% (Selim et al. 2024). The condition is clinically defined by reduced serum concentrations of thyroxine (T4) and triiodothyronine (T3), together with elevated thyroid‐stimulating hormone (TSH). This hormonal imbalance leads to impaired metabolic regulation and dysfunction across multiple organ systems, including the cardiovascular, hepatic, renal, and neurological pathways, as T3 and T4 are essential for cellular metabolism and influence nearly all bodily functions (Haghbin et al. 2024; Olanrewaju et al. 2024; Chauhan and Patel 2024). Autoimmune thyroiditis (Hashimoto's thyroiditis), which involves immune‐mediated destruction of thyroid tissue, is the most common cause of hypothyroidism (Chaker and Papaleontiou 2025). Conventional therapy relies on levothyroxine (LT4) replacement, which effectively restores circulating hormone levels but fails to correct the underlying immune dysregulation. Furthermore, LT4 may induce adverse effects such as palpitations, arrhythmias, and long‐term dependency, especially in patients with cardiac comorbidities (Rossi et al. 2025). These limitations have driven the search for alternative options, particularly plant‐based therapies, which are valued for their relative safety, fewer adverse effects, and cost‐effectiveness. Beyond cost reduction, these natural therapies offer diverse pharmacological benefits—including antioxidative, immunomodulatory, anti‐inflammatory, and hepatoprotective properties—making them an attractive area for research and potential clinical application (Chihomvu et al. 2024; J. K. Paul et al. 2024).

*Withania somnifera* (L.) Dunal, commonly known as Ashwagandha or Indian ginseng, is a renowned adaptogen in ayurvedic medicine (Bhattacharya and Muruganandam 2003). Native to the dry regions of India, North Africa, and the Middle East, this plant in the Solanaceae family has roots that are the primary part used medicinally. Traditionally, the roots are consumed as dried powder mixed with milk, ghee, or honey. The US Food and Drug Administration (FDA) has assigned a Unique Ingredient Identifier (UNII) to *W. somnifera* root, confirming its scientific identity, but this does not equate to Generally Recognized as Safe (GRAS) approval for use in foods and beverages (Mishra et al. 2000). The plant exhibits broad pharmacological activities, including anti‐inflammatory, antioxidant, immunomodulatory, and neuroprotective effects (Vittal and Vinciguerra 2025; Kumar 2026). Preclinical and clinical evidence indicate that its root extracts may normalize thyroid hormone profiles by enhancing T3 and T4 secretion and modulating TSH while demonstrating a favorable safety profile (Chetheer and Ibraheem 2024; Lagah et al. 2024). Early preclinical studies by Panda and Kar (1998,1999) demonstrated that *W. somnifera* root extract could stimulate thyroid hormone production in mice (Sunanda Panda and Kar 1998a; Sunanda Panda and Kar 1998b; S Panda and Kar 1999). More recently, a randomized controlled trial by Sharma et al. (2018) reported that Ashwagandha root extract (600 mg/day for 8 weeks) significantly improved serum TSH, T3, and T4 levels in patients with subclinical hypothyroidism (Sharma et al. 2018). Additional studies have suggested antioxidant‐mediated thyroid protection as a potential mechanism (Abdel‐Wahhab et al. 2019a). However, the molecular mechanisms underlying these beneficial effects are not fully understood. A key knowledge gap exists in characterizing the interaction of its bioactive phytochemicals with key thyroid‐regulating proteins, specifically the thyroid‐stimulating hormone receptor (TSHR) and type 2 iodothyronine deiodinase (D2). The roles of these proteins are critical: TSHR, a G‐protein‐coupled receptor on thyroid follicular cells, mediates thyroid hormone synthesis upon binding TSH, activating cyclic AMP signaling to stimulate T4 and T3 production (Zhang et al. 2024). D2, a selenoprotein enzyme, catalyzes the peripheral conversion of T4 to the more biologically active T3, a process critical for maintaining metabolic homeostasis (Traini et al. 2025). Targeting TSHR and D2 with specific phytochemicals presents a promising therapeutic strategy to enhance thyroid hormone production and activation, potentially addressing the core limitations of conventional LT4 therapy.

**FIGURE 1:**
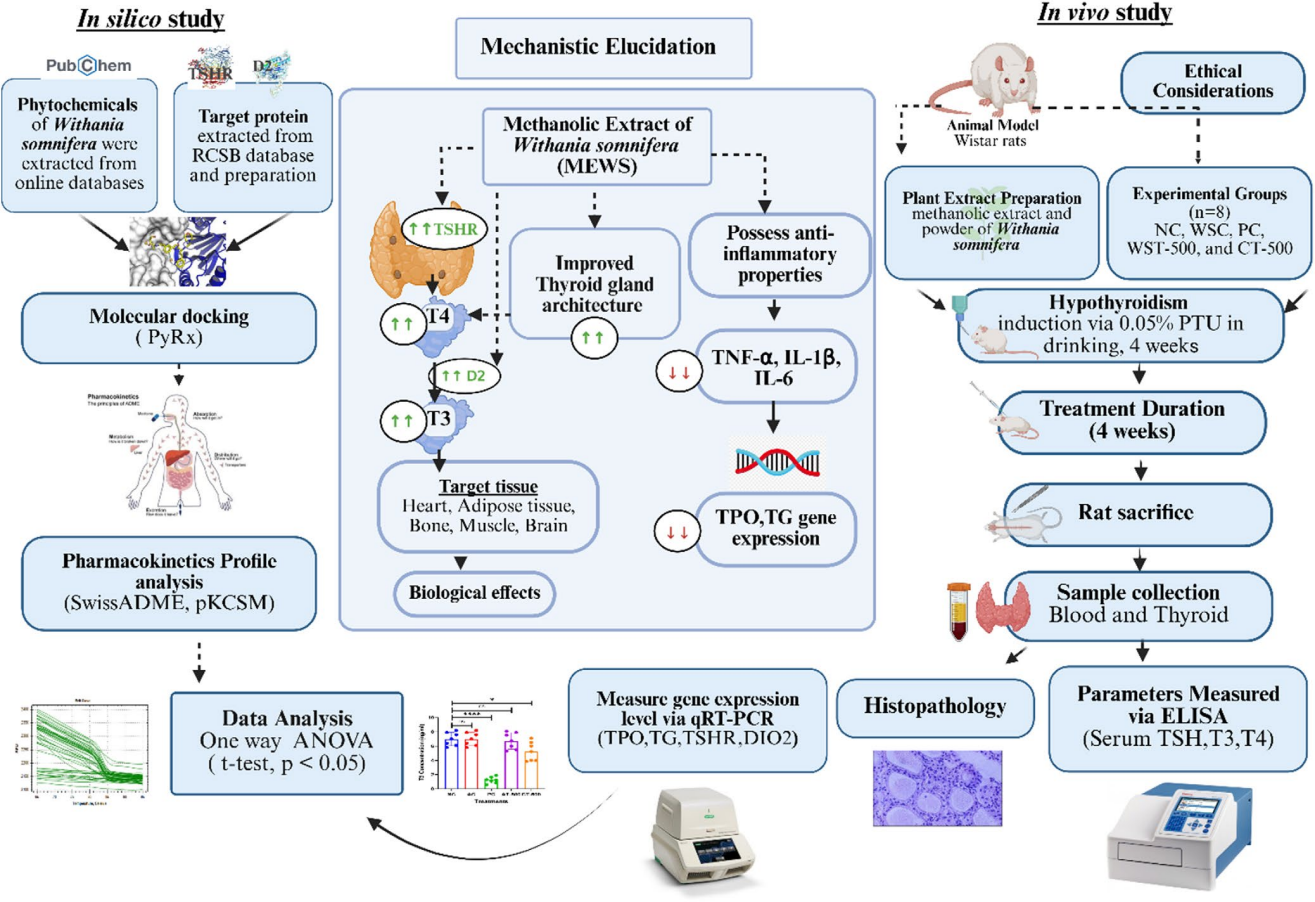
Graphical abstract for the integrated *in silico* and in vivo approach demonstrating the methanolic extract of *Withania somnifera* root ameliorated PTU‐induced hypothyroidism through the regulation of thyroid hormone levels, gene expression, and thyroid histopathology.

To address this knowledge gap, the present study was designed to comprehensively investigate the therapeutic potential and underlying mechanisms of the methanolic extract of *W. somnifera* root (MEWS) in a propylthiouracil (PTU)‐induced hypothyroid rat model, as it reliably induces hypothyroidism by inhibiting thyroid peroxidase, effectively mimicking autoimmune thyroiditis (Suryandari et al. 2024; Ezzat et al. 2024). We hypothesized that MEWS would ameliorate hypothyroidism through dual modulation of thyroid hormone synthesis and peripheral conversion. The specific objectives were to: (i) evaluate the effects of MEWS on serum thyroid hormone profiles (T3, T4, and TSH); (ii) assess histopathological changes in thyroid tissue; (iii) quantify the expression of key thyroid hormone synthesis genes (TPO and TG); and (iv) identify through molecular docking the specific *W. somnifera* phytoconstituents with high binding affinity for TSHR and D2, followed by *in silico* prediction of their drug‐likeness and pharmacokinetic profiles. This integrated experimental strategy, combining in vivo, molecular, and computational approaches, provides a mechanistic framework for understanding *W. somnifera's* thyrotropic effects and supports its evidence‐based application as a nutraceutical intervention for hypothyroidism. This study presents several novel contributions to the field: (i) it is the first to comprehensively *integrate* in vivo thyroid hormone profiling, thyroid histopathology, TPO and TG gene expression analysis, and *in silico* molecular docking against TSHR and D2 targets within a single study on *W. somnifera* ; (ii) it identifies specific phytoconstituents from *W. somnifera* , particularly oxitriptan, as promising lead compounds with high binding affinity to thyroid‐regulating proteins; and (iii) it provides the first predictive pharmacokinetic and drug‐likeness profiles of these *W. somnifera* compounds specifically in the context of thyroid modulation. A graphical overview of this integrated experimental strategy is presented in Figure 1.

## 2 Methods and Materials

### 2.1 Preparation of Plant Extract

The roots of *W. somnifera* were collected from the Medicine Village, Kholbaria, Khamarpara, Natore, Bangladesh, and were validated before being powdered for extraction. The methanolic extract of *W. somnifera* roots was prepared using the maceration technique according to Ghenabzia et al., (Ghenabzia et al. 2023). Here, the powder was mixed with methanol at a ratio of 1:8 (w/v) and shaken regularly. The mixture was then shaken at 130 rpm for 2 h and filtered three times using 125 mm Whatman No. 1 paper. The filtrate was concentrated in a water bath by evaporating methanol at 65°C over 10 days, followed by a final heating step at 80°C for 2 h. The sticky, semi‐solid crude extract was collected and stored at 4°C for further use.

### 2.2 Animal and Experimental Design

Male albino rats (weighing 140–210 g) were acclimatized for 2 weeks under standard laboratory conditions. The animals were kept in a controlled environment with a 12‐h light–dark cycle, a temperature of 25°C ± 2°C, and a relative humidity of 50%–60%. The rats were fed a standard pellet diet and provided with filtered water throughout the trial. Every experimental procedure was carried out in compliance with the ethical standards approved by the Department of Biochemistry and Molecular Biology (BMB) of Mawlana Bhashani Science and Technology University's Ethical Review Committee in Tangail, Bangladesh, with the certificate number MBSTU/BMB/TEST/2/2024/153. All efforts were made to minimize animal suffering and reduce the number of animals used. Animals were randomly allocated to experimental groups using a computer‐generated random number table to ensure unbiased distribution. A total of forty male albino rats were used in the experiment. The rats were randomly divided into five groups, each consisting of eight rats. The NC group (Negative control) consisted of healthy rats receiving a standard diet and water without any treatment. The WSC group ( *W. somnifera* Control) consisted of healthy rats that were orally administered *W. somnifera* methanolic extract (500 mg/kg/day) for 1 month to assess its toxicological and physiological effects (Sultana et al. 2012). PC group (Positive control) comprised hypothyroidism‐induced rats receiving 0.05% (w/v) PTU in drinking water for 6 weeks without any treatment, following established models (Kar et al. 2022; Sahoo et al. 2008). The WST‐500 group ( *W. somnifera* Treatment) consisted of hypothyroid rats treated with a methanolic extract of *W. somnifera* (500 mg/kg/day) for 6 weeks, alongside PTU administration, based on previous reports demonstrating its thyroid‐regulatory effects in hypothyroid rats (Abdel‐Wahhab et al. 2019a). Finally, the CT‐500 group (Combination Therapy) consisted of rats administered PTU (0.05% w/v) in drinking water for 6 weeks, followed by concurrent treatment with *W. somnifera* extract (500 mg/kg/day) for an additional 6 weeks to evaluate synergistic effects. This group was designed to evaluate whether MEWS could provide additional therapeutic benefits when administered after hypothyroidism was fully established, simulating a post‐diagnosis treatment scenario, and to compare its efficacy against the WST‐500 group, where treatment was initiated concurrently with PTU exposure. The dosing volume was 500 mg/kg/day body weight for all groups, calculated based on weekly body weight measurements (Abdel‐Wahhab et al. 2019b). Fresh suspensions were prepared daily, and the gavage procedure was performed by trained personnel to minimize animal stress. For histopathological evaluation, the pathologist was blinded to the treatment groups to ensure unbiased assessment. Investigators conducting biochemical assays (ELISA and qRT‐PCR) were also blinded to group allocation during data collection and analysis. Hypothyroidism was induced by administering PTU, which inhibits thyroid peroxidase activity, mimicking the biochemical and histological features of hypothyroidism (Jena 2015; Kar et al. 2022). The experimental design ensured a comprehensive evaluation of *W. somnifera's* therapeutic potential, both as a standalone treatment and in combination with PTU.

### 2.3 Rat Sacrifice and Sample Collection

Rats were euthanized using chloroform anesthesia, and blood was collected from the hepatic vein under sterile conditions. The blood was allowed to clot on ice for 15–20 min and centrifuged at 5000 rpm for 5 min to separate serum. The serum was aliquoted into sterile Eppendorf tubes and stored at −21°C for biochemical analysis. The thyroid gland was excised, with one part fixed in 10% buffered formalin for histopathology and the other preserved in Tri‐RNA reagent for RNA isolation. The tissue was homogenized using an ultrasound sonicator and then stored on ice after the addition of chloroform to stabilize the RNA. The liver and kidneys were also fixed in 10% buffered formalin (v/v) for further analysis.

### 2.4 Histopathologic Analysis

For histopathological analysis, thyroid gland tissues were carefully excised, cleaned with 0.91% saline solution, and their cellular structure was preserved by fixation in 10% buffered formalin (v/v). Following a sequence of graded ethanol dehydration, the fixed tissues were cleaned in xylene and embedded in paraffin blocks. The tissues embedded in paraffin were cut into sections that were 4–5 μm thick using a Leica model 2165 rotary microtome (Leica Microsystems, Germany). Hematoxylin and Eosin (H&E) staining, a commonly used method in histopathology to visualize cellular and tissue features, was applied to the slices after they had been placed on glass slides (Giri 2020). H&E staining involves the application of hematoxylin, which stains cell nuclei blue, and eosin, which counterstains cytoplasmic and extracellular components pink. This use of differential staining enables the clear visualization of tissue architecture and cellular morphology under a light microscope. The stained slides were examined under light microscopy to assess histological changes in the thyroid gland, including follicular structure, epithelial cell integrity, and signs of inflammation or degeneration.

### 2.5 RNA Isolation, cDNA Synthesis, and Quantitative Real‐Time PCR (qRT‐PCR)

Trizol reagent (FavorPrep Tri‐RNA Reagent) was used to extract total RNA from thyroid tissue samples in accordance with the manufacturer's instructions (Procedure: FavorPrep TM Tri‐RNA Reagent Procedure: Add 1 mL of Tri‐RNA Reagent to 100 Mg Tissue [or Precipitated Blood RNA Viruses from up to 10 ML of Blood or 10^6^ Cultured Cells, or 10 Cm^2^ of Culture Plate] 2. Homogenize Tissue Samples in Tri‐RNA R, n.d.). The quantity and purity of RNA was estimated using Nanodrop. Subsequently, 1 μg of total RNA was reverse‐transcribed into cDNA using the ABscript II cDNA First Strand Synthesis Kit, and RT‐qPCR analysis was performed. Gene expression analysis was performed using the 2^−ΔΔCt^ method, with *β*‐actin as the internal control gene. Using the Primer‐BLAST tool from the National Center for Biotechnology Information (NCBI) (https://www.ncbi.nlm.nih.gov/tools/primer‐blast/), primers were designed for the target genes, thyroid peroxidase (TPO) and thyroglobulin (TG). The primer sequences for TPO (GenBank accession number NM_019353.2) were:

Forward: ATGCTGCCACCGAGATGTCC.

Reverse: GCCATCTTGCTAGGGCTGTG.

The primer sequences for TG (GenBank accession number NM_030988.2) were:

Forward: GGATGAGGCTGGTCAGGAATTG.

Reverse: AGAAGCATTGGAAGCAGAGACG.

The primer sequences for the *β*‐actin gene were:

Forward: CACTGTCGAGTCGCGTCC.

Reverse: TCATCCATGGCGAACTGGTG.

### 2.6 TSH, T3, and T4 Measurement by ELISA

Serum T3, T4, and TSH levels were quantified using commercial ELISA kits (My Biosource Co., Rat Tri‐iodothyronine (T3) and Thyroxine (T4) ELISA Kit, and Rat Thyroid Stimulating Hormone (TSH) ELISA Kit, respectively). The assays were performed according to the manufacturer's instructions. Briefly, for each assay, standards or samples were added to designated wells and incubated with the appropriate conjugate. Following incubation and subsequent washes, substrate solutions were added, and the colorimetric reaction was developed. After the reaction was halted, a Thermo Scientific Multiscan FC microplate reader was used to measure the optical density of each well at 450 nm. The concentration of T3, T4, and TSH in the samples was determined by interpolating the measured optical densities onto standard curves generated from known concentrations of the respective analytes.

### 2.7 Phytochemicals, Protein Retrieval, and Preparation

A comprehensive literature review identified twenty‐one phytochemicals derived from LC/MS/MS analysis of the methanolic extract of *W. somnifera* (ashwagandha) roots (Abdel‐Wahhab et al. 2019a). L‐Thyroxine is used as a control drug. Three‐dimensional structures and SDF files for all phytochemicals and the selected drug were obtained from the publicly accessible PubChem database (https://pubchem.ncbi.nlm.nih.gov/) (Kim et al. 2016). Following the phytochemical acquisition, target protein structures, namely the thyroid‐stimulating hormone receptor (TSHR, PDB ID: 2XWT) and D2 iodothyronine deiodinase (PDB ID: 9H48), were obtained in PDB format from the RCSB Protein Data Bank (https://www.rcsb.org/structure/) (Oliveira et al. 2017). Before docking studies, protein preparation was conducted to optimize binding efficiency at minimal energy conformations. Utilizing BIOVIA Discovery Studio Visualizer v19.1.0.18287, extraneous components, including non‐essential protein chains, water molecules, heteroatoms, and existing ligands, were removed from the raw protein structures. Subsequent energy minimization was performed using the Swiss PDBViewer (https://spdbv.unil.ch/), a crucial step in refining protein structures and enhancing potential ligand‐binding interactions (Guex and Peitsch 1997).

### 2.8 Molecular Docking

Molecular docking was performed using PyRx software for visualization purposes, following approaches similar to those described by Kar et al. in their study on caffeic acid modulation of PTU‐induced hypothyroidism (Kar et al. 2022). Grid boxes encompassing the active sites of each protein were generated as follows: for TSHR (PDB ID: 2XWT), the grid center was defined at x: y: z = 16.5698: −19.693: 6.5796 with dimensions of 25 Å in each x, y, and z axis; for iodothyronine deiodinase (PDB ID: 9H48), the grid center was set at x: y: z = 13.9289: −1.5395: 6.0568 with dimensions of 48.4436, 44.0350, and 55.1029 Å in the x, y, and z axes, respectively. An exhaustiveness value of 50 was implemented to ensure a comprehensive sampling of potential binding poses for each ligand. Complexes demonstrating the most negative docking scores, indicative of the strongest binding affinities, were prioritized as the most promising candidates. Protein‐ligand interactions were then analyzed and visualized using BIOVIA Discovery Studio Visualizer (Shuvo et al. 2025).

### 2.9 Drug Likeness, Pharmacokinetics, and Toxicity Properties Prediction

Drug likeness was evaluated using Lipinski's Rule of Five via the SwissADME (http://www.swissadme.ch/) web server (Lipinski et al. 1997). This evaluation is crucial for predicting the pharmacokinetic properties (absorption, distribution, metabolism, and excretion) of small‐molecule candidates. The toxicity of selected phytochemicals was assessed using the pKCSM (https://biosig.lab.uq.edu.au/pkcsm/) web server to analyze potential toxicological risks of these phytochemicals.

### 2.10 Statistical Analysis

Data were analyzed using GraphPad Prism version 8.0 (GraphPad Software, San Diego, CA, USA). Results are expressed as mean ± SEM. All data met these assumptions (*p* > 0.05), justifying the use of parametric tests. Group differences were evaluated by one‐way ANOVA, followed by Tukey's multiple comparison test for post hoc analysis. Each experimental group consisted of 8 participants. Exact *p*‐values are reported in the figure legends as follows: ****p < 0.0001, ***p < 0.001, **p < 0.01, *p < 0.05, with “ns” indicating non‐significant (*p* > 0.05). Statistical significance was set at *p* < 0.05.

## 3 Result

### 3.1 Effects of *Withania somnifera* Extract on Thyroid Gland Morphology and Weight

**FIGURE 2:**
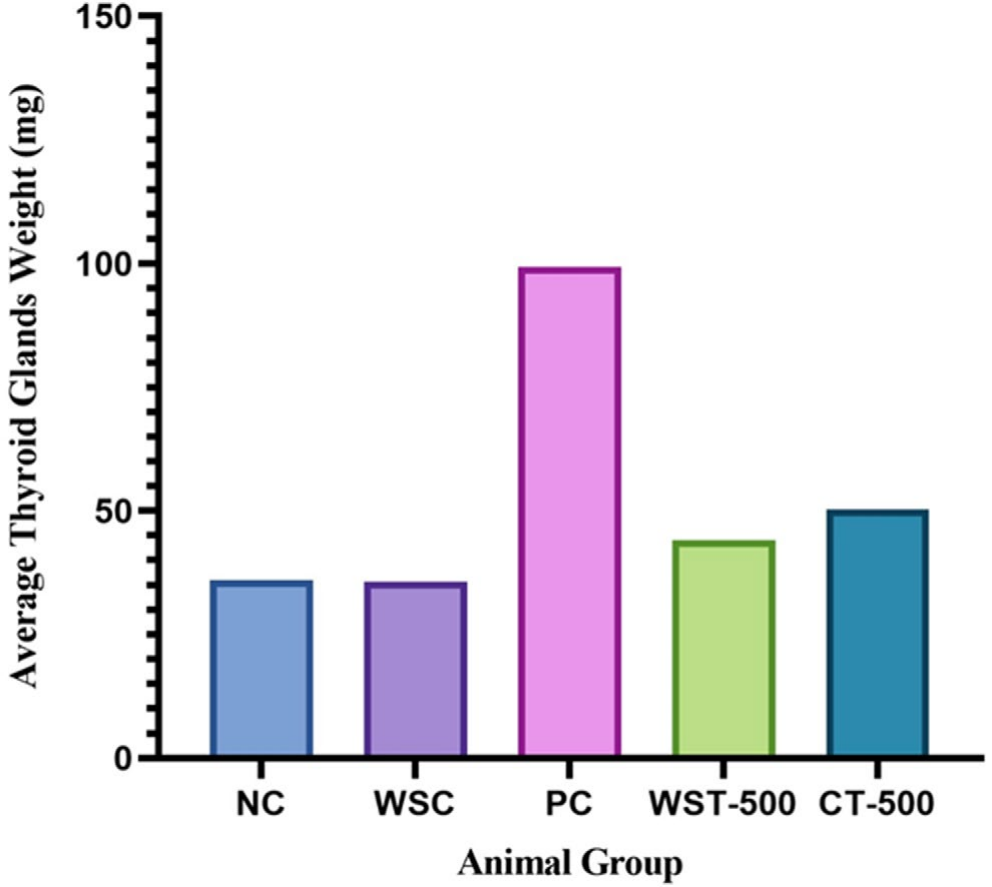
*Thyroid gland enlargement and therapeutic impact of Withania somnifera extract in experimental models*. The thyroid gland weight was significantly increased by approximately 2.8‐fold in the hypothyroid group (positive control) compared to the negative control group. Treatment with MEWS at 500 mg/kg/day reduced thyroid gland weight by 2.3‐fold, a more significant reduction than the negative control. A synergistic treatment (CT‐500) further reduced it to 2‐fold, a more significant reduction than the negative control. Here, NC (negative control), WSC ( *W. somnifera* control), PC (positive control), WST‐500 ( *W. somnifera* treatment 500 mg/kg/day), CT‐500 (combination therapy (MEWS + PTU)), MEWS (m*ethanolic extract* of *Withania somnifera* ). Data are presented as mean ± SEM (*n* = 8 per group).

Induction of hypothyroidism with PTU resulted in a significant increase in absolute thyroid gland weight in the positive control (PC) group compared to the negative control (NC) group (2.8‐fold increase, Figure 2, Table S1). Treatment with *W. somnifera* extract (WST‐500) significantly attenuated this effect, reducing thyroid weight to 2.3 times that of the NC. The combination treatment (CT‐500) demonstrated a more pronounced effect, further reducing the weight by 2.0‐fold relative to NC. A similar pattern of normalization was observed for relative thyroid weight.

These findings demonstrate a clear dose‐ and treatment‐dependent effect, suggesting that MEWS alleviates PTU‐induced thyroid hypertrophy and supports restoration of glandular homeostasis.

### 3.2 Histopathological Changes in the Thyroid Gland Following *Withania somnifera* Treatment

**FIGURE 3:**
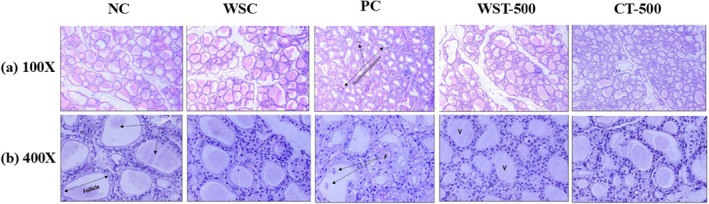
*Histopathological analysis of thyroid gland stained with H&E (100X and 400X)*: The NC group exhibited normal follicular architecture with colloid‐filled lumens. The WSC group displayed a similar morphology to NC. PC group (hypothyroid) showed severe disruptions, including disorganized follicles, irregular colloids, vacuolation, fibrosis, and vascular congestion. The WST‐500 group showed marked improvement, with reduced fibrosis and proliferation, though vacuolation persisted. The CT‐500 groups demonstrated near‐normal thyroid structure with minimal fibrosis and vacuolation, highlighting the protective effects of *Withania somnifera* extract. Here, NC (Negative Control), WSC ( *W. somnifera* Control), PC (Positive Control), WST‐500 ( *W. somnifera* Treatment 500 mg/kg/day), CT‐500 (Combination Therapy [MEWS + PTU]), MEWS (Methanolic Extract of *Withania somnifera* ). Representative images are shown, V = Vacuolation; F = Fibrosis.

The histological features of PTU‐induced hypothyroidism were examined using H&E staining. The NC and WSC groups exhibited normal thyroid morphology, characterized by well‐organized follicles and colloid‐filled lumens (Figure 3). In contrast, the PC group exhibited severe architectural alterations, including follicular disorganization, vacuolation, fibrosis, and vascular congestion.

Treatment with WST‐500 partially improved thyroid morphology by restoring follicular organization, reducing fibrosis, and enhancing colloid retention; however, mild vacuolation persisted. Notably, the CT‐500 group exhibited near‐complete restoration of thyroid architecture, characterized by minimal fibrosis and vacuolation. These improvements were more pronounced than those in the WST‐500 group, underscoring the synergistic protective effect of combination therapy and reinforcing the therapeutic efficacy of MEWS.

### 3.3 Modulation of Thyroid Hormone Levels by *Withania somnifera* Extract: ELISA‐Based Analysis

**FIGURE 4:**
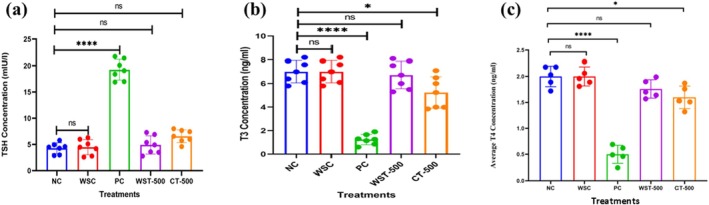
*Modulation of thyroid hormone levels by Withania somnifera methanolic extract*. Serum T3, T4 (ng/ml), and TSH (mIU/l) levels were measured across experimental groups. For TSH levels (a), the positive control group (PC) displayed markedly elevated concentrations compared to the negative control group (NC) (****p < 0.0001). Treatment with *W. somnifera* extract (WST‐500) and combination therapy (CT‐500) significantly reduced TSH levels, nearing those of the negative control group (NC) (****p < 0.0001 vs. PC; ns vs. NC indicating no significant difference). For T3 (b) and T4 (c) levels, the NC group exhibited normal values, while the hypothyroid positive control group (PC) showed significantly reduced levels (****p < 0.0001 vs. NC). Treatment groups (WST‐500 and CT‐500) demonstrated significantly improved T3 and T4 levels compared to PC **(****p < 0.0001 for WST‐500 vs. PC; *p < 0.001 for CT‐500 vs. PC), indicating partial restoration of thyroid homeostasis. Data are presented as means ± SEM (*n* = 8 per group). Statistical significance: ****p < 0.0001, ***p < 0.001, **p < 0.01, *p < 0.05; “ns” indicates non‐significant (*p* > 0.05). NC (Negative Control), WSC ( *W. somnifera* Control), PC (Positive Control), WST‐500 ( *W. somnifera* Treatment 500 mg/kg/day), CT‐500 (Combination Therapy: MEWS + PTU), MEWS (Methanolic Extract of *W. somnifera* ).

ELISA confirmed the induction of hypothyroidism in the PC group, reflected by significantly elevated TSH (19.08 ± 1.1 mIU/L vs. 4.29 ± 0.6 mIU/L in NC; *p* < 0.001) and markedly reduced T3 (1.26 ± 0.38 vs. 7.00 ± 0.51 ng/mL; *p* < 0.0001) and T4 (0.50 ± 0.09 vs. 2.00 ± 0.15 μg/dL; *p* < 0.0001) (Figure 4; Tables S2–S4).

Treatment with WST‐500 effectively reversed these alterations, reducing TSH levels (4.92 ± 0.83 mIU/L; *p* < 0.0001 vs. PC) while restoring T3 (6.72 ± 0.51 ng/mL; *p* < 0.0001 vs. PC) and T4 (1.80 ± 0.14 μg/dL; *p* < 0.0001 vs. PC) levels to near‐normal levels (ns vs. NC for both T3 and T4, indicating no significant difference). CT‐500 treatment also improved hormone profiles (TSH: 6.58 ± 0.89 mIU/L, *p* < 0.0001 vs. PC T3: 5.24 ± 0.73 ng/mL, *p* < 0.001 vs. PC; T4: 1.50 ± 0.18 μg/d, *p* < 0.001 vs. PC), though recovery of T3 and T4 was slightly less complete compared to WST‐500 (*p* < 0.05 for WST‐500 vs. CT‐500). Tukey's post hoc analysis confirmed significant differences between PC and both treatment groups. Overall, these findings demonstrate that MEWS restores thyroid hormone balance in a treatment‐dependent manner.

### 3.4 Gene Expression Analysis of Thyroid Hormone‐Regulating Genes in Hypothyroid Rats

**FIGURE 5:**
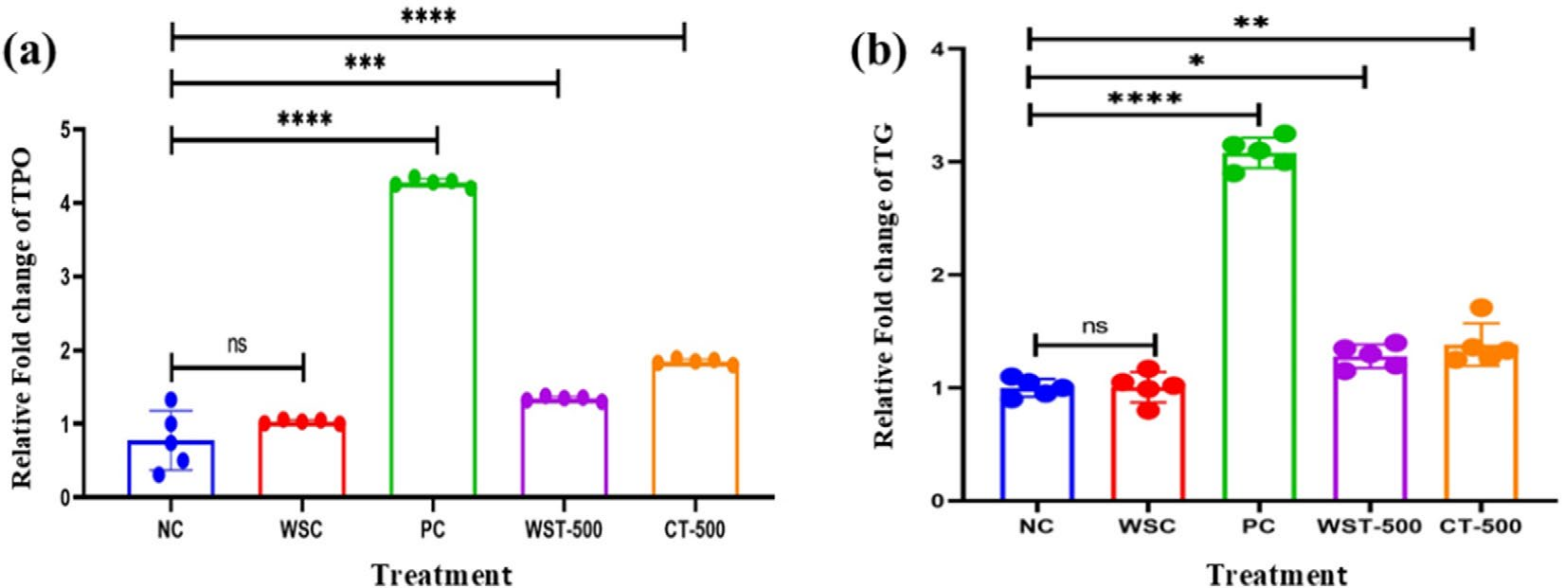
*Effects of Withania somnifera methanolic extract on the gene expression of TPO and TG in hypothyroid rats*. RT‐PCR analysis revealed significant upregulation of TPO (4.28 ± 0.32‐fold, *p* < 0.0001 vs. NC) and TG (3.10 ± 0.60‐fold, *p < 0.001 vs. NC) gene expression in the hypothyroid positive control group (PC) compared to the negative control group (NC). Treatment with *W. somnifera* extract (WST‐500) and combined therapy (CT‐500) markedly reduced these expressions. TPO expression levels decreased to **1.35 **±** 0.29 RFC (**p < 0.0001 vs. PC; ns vs. NC) and 1.85 ± 0.38 RFC (*p* < 0.01 vs. PC) in the WST‐500 and CT‐500 groups, respectively (a). Similarly, TG expression was reduced to 1.30 ± 0.69 RFC (*p* < 0.01 vs. PC; ns vs. NC) and 1.36 **±** 0.31 RFC (*p* < 0.01 vs. PC). (b) Data are means ± SEM (*n* = 8 per group). Statistical significance: ****p < 0.0001, ***p < 0.001, *p < 0.*01, p* < 0.*05* vs. respective control (NC); *“ns” indicates no significant difference* (*p* > 0.05). NC (negative control), WSC ( *W. somnifera* control), PC (positive control), WST‐500 ( *W. somnifera* treatment 500 mg/kg/day), CT‐500 (combination therapy: MEWS + PTU), MEWS (methanolic extract of *W. somnifera* ).

qPCR analysis revealed significant transcriptional dysregulation induced by PTU (Figure 5). In the PC group, TPO expression increased ~4.28 ± 0.32‐fold (*p* < 0.0001 vs. NC) and TG ~3.10 ± 0.60‐fold (*p* < 0.001 vs. NC) compared to NC. Treatment with WST‐500 markedly attenuated this upregulation, normalizing TPO (1.35 ± 0.29 RFC; *p* < 0.0001 vs. PC; ns vs. NC) and TG (1.30 ± 0.69 RFC; *p* < 0.01 vs. PC; ns vs. NC). CT‐500 also reduced expression levels (TPO: 1.85 ± 0.38 RFC, *p* < 0.01 vs. PC; TG: 1.36 ± 0.31 RFC; p < 0.01 vs. PC).

This normalization of gene expression is consistent with improvements in thyroid morphology and hormone balance, suggesting that MEWS exerts therapeutic benefits at both the molecular and systemic levels.

### 3.5 Molecular Docking Analysis of *Withania somnifera* Phytochemicals With Thyroid Hormone‐Related Targets

Molecular docking was performed to predict the optimal intermolecular interactions between the target proteins and small molecular drug candidates, as measured by binding affinities and interaction poses. The binding potential of selected *W. somnifera* phytochemicals with two thyroid hormone‐related proteins: the thyroid‐stimulating hormone receptor (TSHR, PDB ID: 2XWT) and Type 2 iodothyronine deiodinase (D2, PDB ID: 9H48). Binding affinities (ΔG, kcal/mol) are presented in Table 1. More negative ΔG values correspond to stronger predicted binding. Among the screened compounds, caftaric acid exhibited the most favorable predicted binding energies (−6.7 kcal/mol for both TSHR and D2), followed by 6‐benzylaminopurine and tryptophylglycine (−6.3 to −6.6 kcal/mol). Oxitriptan also demonstrated moderate predicted binding affinity, consistently outperforming the reference ligand, L‐thyroxine, which bound TSHR at −5.3 kcal/mol and D2 at −6.0 kcal/mol.

**TABLE 1:** Binding affinity of top *Withania somnifera* ‐derived phytochemicals with TSHR and D2 iodothyronine deiodinase enzyme.

Phytochemicals Name (PubChem ID)	Binding Energy (ΔG = kcal/mol) to Selected Proteins	
	TSHR (PDB ID: 2XWT)	D2 Iodothyronine deiodinase (PDB ID: 9H48)
Caftaric acid (6440397)	−6.7	−6.7
6‐Benzylaminopurine (62389)	−6.3	−6.5
Tryptophylglycine (97054)	−6.5	−6.6
Oxitriptan (439280)	−5.8	−6.2
6‐Hydroxy‐4‐methyl‐coumarin (75409)	−6.1	−5.6
6‐Bromoquinolin‐2 (1H)‐one (12378943)	−5.6	−5.4
Caffeic Acid (689043)	−6	−6.1
Epinephrine (5816)	−5.8	−5.5
L‐thyroxine (control)	−5.3	−6.0

*Note:* Docking scores (ΔG, kcal/mol) reflect binding affinities of *W. somnifera* phytochemicals with thyroid proteins. Negative ΔG indicates stable interactions, with caftaric acid, 6‐benzylaminopurine, and tryptophylglycine showing stronger affinities than L‐thyroxine, suggesting therapeutic potential.

These results suggest a strong potential for several *W. somnifera* phytochemicals to act as modulators of TSHR and D2, forming stable complexes that could directly influence thyroid hormone regulation in hypothyroidism. However, these computational predictions require experimental validation through in vitro and in vivo studies to confirm actual biological activity.

#### 3.5.1 Protein‐Ligand Interactions Interpretation After Molecular Docking Analysis/Binding Affinity Analysis

**FIGURE 6:**
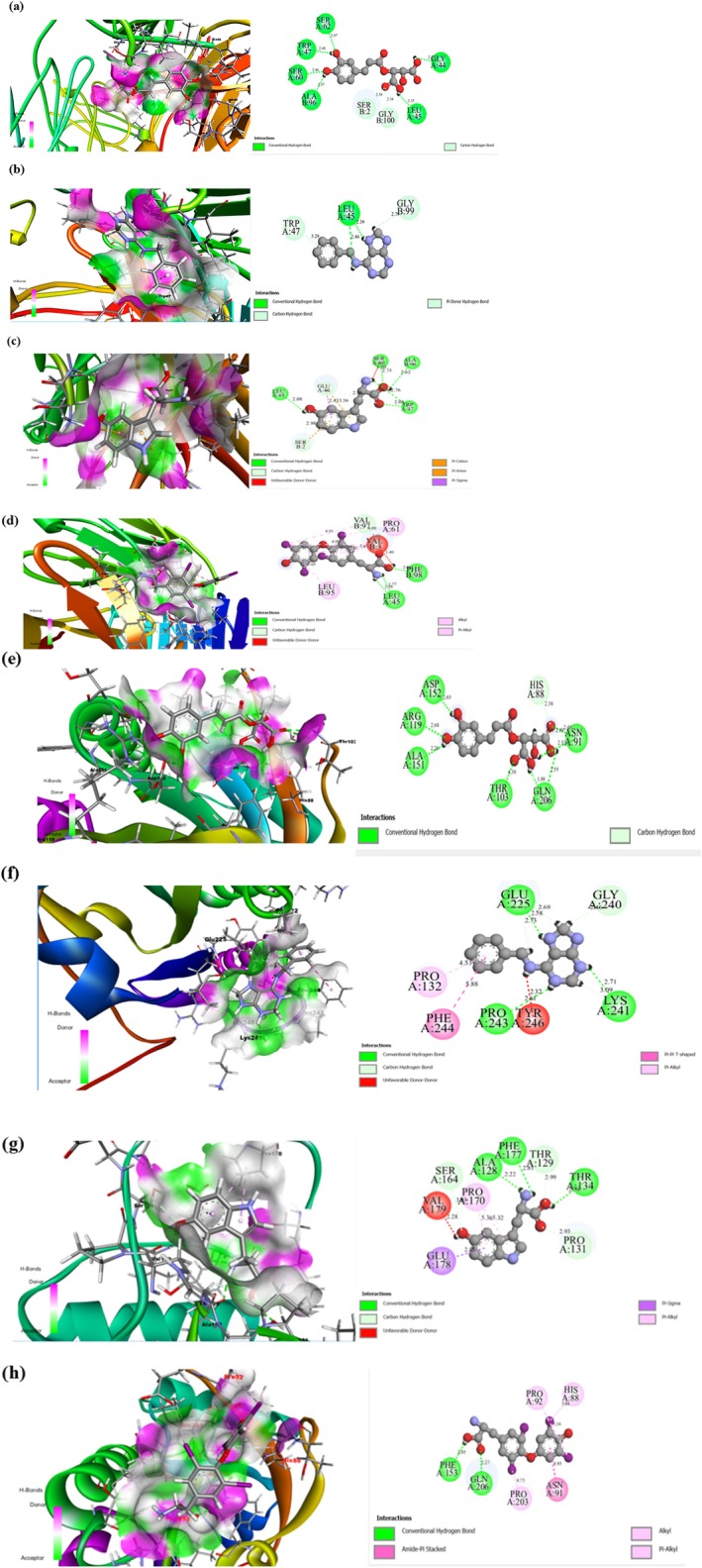
*Visualization of binding modes of selected compounds with target proteins*. (a), (b) and (c) display the binding interactions of caftaric acid (6440397), 6‐benzylaminopurine (62389), and oxitriptan (439280), respectively, along with the control drug thyroxine (5819) in (d), with the TSH receptor protein (PDB ID: 2XWT). Similarly, (e), (f), and (g) illustrate the binding of caftaric acid, 6‐benzylaminopurine, and oxitriptan, respectively, while (h) shows the binding of thyroxine with the D2 iodothyronine deiodinase protein (PDB ID: 9H48).

The specific amino acid residues contributing to predicted ligand–protein interactions were further characterized (Table 2, Figure 6). For TSHR, caftaric acid showed potential interaction through multiple hydrogen bonds with LEU45, SER62, TRP47, SER60, and GLY44 (Chain A), as well as ALA96 (Chain B), and predicted hydrophobic interaction with SER2 and GLY100 (Chain B). 6‐benzylaminopurine interacted primarily with LEU45 (Chain A) via hydrogen bonding and formed potential hydrophobic bonds with TRP47 (Chain A) and GLY99 (Chain B). Oxitriptan is predicted to form hydrogen bonds with LEU45, SER60, TRP47, and ALA96 (Chain B), and hydrophobic interactions with GLU46 (Chain A) and SER2 (Chain B). The control ligand, L‐thyroxine, also interacted with LEU45 (Chain A), along with PHE98 (Chain B), while forming hydrophobic contacts with PRO91 (Chain A), and VAL3, LEU95, and VAL97 (Chain B). Notably, LEU45 emerged as a common predicted interaction site across caftaric acid, 6‐benzylaminopurine, oxitriptan, and L‐thyroxine, suggesting this residue may play a critical role in TSHR ligand binding.

**TABLE 2:** Non‐bonding interactions between TSHR (Thyroid Stimulating Hormone Receptor) and iodothyronine deiodinase with the top 3 phytochemical compounds and control drug.

Target protein	Ligands (PubChem CID)	Binding affinity (kcal/mol)	Amino acids involved in interactions	
			Hydrogen bond interaction	Hydrophobic bond interaction
TSHR (PDB ID: 2XWT)	Caftaric acid (6440397)	−6.7	LEU (A) 45, SER (A) 62, TRP (A) 47, SER (A) 60, GLY (A) 44, ALA (B) 96	SER (B) 2, GLY (B) 100
	6‐Benzylaminopurine (62389)	−6.3	2X LEU (A) 45	TRP (A) 47, GLY (B) 99
	Oxitriptan (439280)	−5.8	LEU (A) 45, SER (A) 60, TRP (A) 47, ALA (B) 96	GLU (A) 46, SER (B) 2
	L‐thyroxine (Control) (5819)	−5.3	LEU(A) 45, PHE(B) 98	PRO (A) 91, VAL (B) 3, LEU (B) 95, VAL (B) 97
D2 Iodothyronine deiodinase (PDB ID: 9H48)	Caftaric acid (6440397)	−6.7	GLN 206, ASP 152, ARG 119, ALA 151, THR 103, ASN 91	HIS 88
	6‐Benzylaminopurine (62389)	−6.5	GLU 225, PRO 243, LYS 241	PRO 132, PHE 244, TYR 246, GLY 240
	Oxitriptan (439280)	−6.2	ALA 128, PHE 177, THR 134	SER 164, PRO 170, VAL 179, GLU 178, THR 129, PRO 131
	L‐thyroxine (5819) (Control)	−6	GLN 206, PHE 153	PRO 92, HIS 88, PRO 203, ASN 91

*Note:* Docking simulations identified binding affinities and key residues. Hydrogen and hydrophobic interactions stabilize the complexes, with caftaric acid showing broad hydrogen bonding to TSHR and D2, potentially modulating thyroid hormone regulation.

For D2, caftaric acid formed predicted hydrogen bonds with GLN206, ASP152, ARG119, ALA151, THR103, and ASN91, as well as a potential hydrophobic contact at HIS88. 6‐benzylaminopurine showed predicted interaction with GLU225, PRO243, and LYS241 via hydrogen bonds, and with PRO132, PHE244, TYR246, and GLY240 through predicted hydrophobic interactions. Oxitriptan was predicted to form hydrogen bonds with ALA128, PHE177, and THR134, and hydrophobic interaction with SER164, PRO170, VAL179, GLU178, THR129, and PRO131. L‐thyroxine shared common predicted binding motifs with caftaric acid at GLN206 and HIS88, highlighting potential structural overlap in their interaction with D2.

The detailed characterization of these potential hydrogen bonds and hydrophobic interactions provides preliminary information for structure–activity relationship studies and rational drug design, offering hypotheses for how MEWS‐derived compounds may stabilize key protein–ligand complexes to influence thyroid hormone synthesis and metabolism. These *in silico* predictions serve as a foundation for future experimental validation studies.

### 3.6 Prediction of Physicochemical and Drug‐Likeness Properties

The drug‐likeness of a compound explains its potential as a drug molecule candidate. The physicochemical properties and drug‐likeness of caftaric acid, 6‐benzylaminopurine, and oxitriptan were compared with L‐thyroxine (Table 3). The bio‐pharmacological activities of novel candidates are often evaluated using Lipinski's Rule of Five (Kowalska et al. 2018). All three phytochemicals met this criterion, predicting strong potential for oral bioavailability. However, caftaric acid violated Ghose, Veber, and Egan criteria and triggered a PAINS alert, suggesting potential limitations in its developability despite strong docking performance. In contrast, 6‐benzylaminopurine and oxitriptan exhibited no violations or alerts, indicating more favorable drug‐like profiles based on these computational predictions. The fact that these top hit compounds showed no Lipinski violations supports their predicted drug‐likeness properties, with oxitriptan emerging as an up‐and‐coming candidate for further investigation.

**TABLE 3:** Physicochemical and drug‐likeness properties of selected 3 phytochemicals and FDA‐approved drug L‐thyroxine.

Parameters	Caftaric acid	6‐Benzylaminopurine	Oxitriptan	L‐thyroxine (Control)
**Physiochemical**				
Chemical formula	C_13_H_12_O_9_	C_12_H_11_N_5_	C11H12N2O3	C_15_H_11_I_4_NO_4_
Molecular weight (g/mol)	312.23	225.25	220.22	776.87
H‐Bond Acceptor	9	3	4	5
H‐Bond Donor	5	2	4	3
Fraction Csp3	0.15	0.08	0.18	0.13
Rotatable bonds	7	3	3	5
Topological polar surface area (*Å* ^2^)	161.59	66.49	99.34	92.78
**Lipophilicity Log Po/w**				
XLOGP3	0.06	1.75	−1.17	2.36
MLOGP	−0.70	1.20	−2.22	2.35
Consensus (Log Po/w)	−0.23	1.54	−0.26	3.54
**Drug Likeness**				
Bioavailability Score	0.11	0.55	0.55	0.55
Lipinski	Yes; 0 violation	Yes; 0 violation	Yes; 0 violation	Yes; 1 violation: MW > 500
Ghose	No; 1 violation WLOGP < −0.4	Yes	Yes	No; 1 violation: MW > 480
Veber	No; 1 violation TPSA> 140	Yes	Yes	Yes
Egan	No; 1 violation TPSA> 131.6	Yes	Yes	Yes
PAINS	No; 1 alert: catechol_A	0 alert	0 alert	0 alert
Drug likeness	Yes	Yes	Yes	Yes

*Note:* All three phytochemicals satisfied major drug‐likeness criteria. L‐thyroxine violates Lipinski's MW rule but is clinically effective, highlighting the predictive nature of these filters.

### 3.7 Pharmacokinetics Properties Prediction

In the drug discovery process, pharmacokinetics (PK) plays a vital role in exploring lead compounds by optimizing their ADMET properties. *In silico* ADMET profiling is crucial for every biomolecule before its biotransformation into a drug, as these properties determine the pharmacological action on the body and must be suitable to pass clinical trials (Wang and Urban 2004). Computational predictions are summarized in Table 4.

**TABLE 4:** Pharmacokinetics profile of the selected three potential drug candidates derived from SwissADME and pKCSM web servers.

Parameters	Caftaric acid	6‐Benzylaminopurine	Oxitriptan	L‐thyroxine (control)
**Absorption**				
Water solubility (log mol/L)	−2.541	−2.789	−2.891	−2.891
Gastrointestinal (% Absorbed)	9.399 (Low)	93.256 (High)	55.592 (High)	58.699 (High)
Log Kp (skin permeation) cm/s	−8.16	−6.43	−2735	−9.36
**Distribution**				
BBB permeability (log BB)	−1.233	0.474	−0.665	−1.433
CNS permeation (Log PS)	−3.93	−3.3	−2.828	−2.279
Volume of distribution (human) (log L/kg)	−0.919	0.456	0.007	−0.069
**Metabolism**				
CYP2D6 substrate	No	No	No	No
CYP3A4 substrate	No	No	No	No
P‐gp substrate	No	Yes	No	Yes
CYP1A2 inhibitor	No	Yes	No	No
CYP2C19 inhibitor	No	No	No	No
CYP2C9 inhibitor	No	No	No	No
CYP2D6 inhibitor	No	No	No	No
CYP3A4 inhibitor	No	Yes	No	No
**Excretion**				
Total clearance (log mL/min/kg)	0.449	0.933	0.71	−0.387
Renal OCT2 substrate	No	No	No	No
**Toxicity properties prediction**				
Ames toxicity	No	Yes	No	No
hERG I inhibitor	No	No	No	No
hERG II inhibitor	No	Yes	No	No
Maximum tolerated dose (human)	0.926	0.875	0.723	0.505
Rat acute toxicity (LD50, mol/kg)	2.174	2.395	2.413	2.451
Hepatotoxicity	No	Yes	No	No
Skin sensitization	No	No	No	No
*T. pyriformis* toxicity	0.285	0.285	0.285	0.285
Minnow toxicity	4.872	2.58	2.221	−0.734

*Note:* ADMET properties were predicted using SwissADME and pkCSM. Caftaric acid was non‐toxic and may benefit from formulation strategies to improve absorption, while 6‐benzylaminopurine showed high absorption, though its potential toxicity warrants further safety evaluation.

In terms of predicted absorption, PK models indicate that an absorption value of less than 30% suggests a drug candidate is poorly absorbed. Caftaric acid demonstrated poor predicted gastrointestinal uptake (9.4%), while 6‐benzylaminopurine and oxitriptan showed high predicted absorption (> 55%), comparable to L‐thyroxine.

Regarding predicted distribution, 6‐benzylaminopurine displayed the highest estimated blood–brain barrier (BBB) penetration and CNS permeability. Drugs having a logBB value > 0.3 are considered highly permeable to the BBB; 6‐benzylaminopurine (logBB: 0.474) meets this criterion, suggesting potential for central effects, which may not be desirable for a peripheral thyroid treatment and would need to be carefully evaluated in future studies.

Metabolic predictions indicated that none of the compounds were predicted substrates for CYP2D6 or CYP3A4, two primary isozymes responsible for drug metabolism. However, 6‐benzylaminopurine was identified as a potential substrate for P‐gp and a predicted inhibitor of CYP1A2/3A4, suggesting possible drug–drug interactions that warrant further investigation.

Toxicity predictions revealed potential concerns for 6‐benzylaminopurine, including predicted Ames mutagenicity, hERG II inhibition, and hepatotoxicity. Computational toxicity testing is a valuable and cost‐effective tool in preclinical development for identifying such liabilities early. In contrast, oxitriptan demonstrated a balanced predicted ADMET profile, characterized by good absorption, no major toxicity alerts, and no mutagenicity, thereby emerging as the most promising lead compound for further experimental validation.

## 4 Discussion

Hypothyroidism is a prevalent endocrine disorder characterized by insufficient thyroid hormone production, leading to systemic metabolic dysfunction and clinical manifestations such as fatigue, weight gain, and cold intolerance. Biochemically, it presents with elevated serum TSH and reduced T3 and T4, reflecting impaired thyroid hormone biosynthesis and compensatory pituitary feedback (Chaker and Papaleontiou 2025). Although levothyroxine replacement remains the standard therapy, it has limitations, including variable absorption, dose adjustment challenges, and inability to address autoimmune pathology, leaving many patients with persistent symptoms such as palpitations, arrhythmias, and long‐term dependency, especially in patients with cardiac comorbidities (Rossi et al. 2025). These limitations underscore the need for complementary strategies that not only restore hormonal balance but also protect thyroid structure and function, which may have relevance to various forms of thyroid dysfunction, including those with inflammatory components.

At the molecular level, thyroid peroxidase (TPO) and thyroglobulin (TG) are essential for thyroid hormone biosynthesis. Dysregulation of these genes is implicated in hypothyroidism and autoimmune thyroiditis (Kochman et al. 2021; Vargas‐Uricoechea 2023). PTU, a classical antithyroid drug, induces hypothyroidism by inhibiting TPO activity, thereby suppressing thyroid hormone synthesis and disrupting glandular architecture (Kar et al. 2022; Park and Lee 2021). Upregulation of TPO and TG following PTU exposure represents a compensatory biosynthetic response to impaired hormone synthesis. While similar gene expression patterns have been observed in autoimmune thyroiditis (Balkrishna et al. 2024). It should be noted that the PTU‐induced model primarily reflects chemical inhibition of thyroid function rather than immune‐mediated destruction. Nevertheless, the gene expression changes observed may have relevance for understanding thyroid biosynthetic regulation in various pathological contexts.

Our findings demonstrate that the methanolic extract of *W. somnifera* (MEWS) significantly alleviated PTU‐induced hypothyroidism. MEWS reduced thyroid hypertrophy, restored follicular organization, and normalized T3 and T4 levels while lowering elevated TSH. The concurrent increase in T4 levels and the normalization of serum TSH suggest that MEWS may enhance the peripheral conversion of T4 to the more active T3, a hypothesis supported by our *in silico* docking results with D2. At the transcriptional level, it attenuated PTU‐induced upregulation of TPO and TG, suggesting restoration of biosynthetic regulation. Histopathological analysis further confirmed tissue protection, showing reduced fibrosis, vascular congestion, and follicular vacuolation. These results indicate that MEWS exerts multi‐level protective effects encompassing biochemical, genetic, and morphological domains, consistent with recent preclinical evidence for *W. somnifera* in endocrine disorders (Gaurav et al. 2023; S. Paul et al. 2021).

Our findings are consistent with and extend previous research on *W. somnifera's* thyrotropic effects. Early studies by Panda and Kar (1998, 1999) first reported that *W. somnifera* root extract increased serum T4 levels in mice, though the mechanisms remained unexplored (Sunanda Panda and Kar 1998a; Sunanda Panda and Kar 1998b; S Panda and Kar 1999). More recently, a clinical trial by Sharma et al. (2018) demonstrated that *W. somnifera* root extract (600 mg/day for 8 weeks) significantly improved TSH, T3, and T4 levels in patients with subclinical hypothyroidism, with 79.2% of treated patients showing normalized TSH compared to 10.7% in the placebo group (Sharma et al. 2018; Abdel‐Wahhab et al. (2019b)) further showed that *W. somnifera* extract protected against PTU‐induced hypothyroidism in rats, attributing effects to antioxidant activity (Abdel‐Wahhab et al. 2019a). Our study advances these findings by: (i) providing the first evidence of TPO and TG gene expression modulation by *W. somnifera* ; (ii) identifying specific phytochemicals (oxitriptan) with predicted binding to TSHR and D2; and (iii) integrating histopathological, biochemical, and molecular data within a single comprehensive framework. Together, these data and MEWS's modulation of TPO/TG gene expression may indicate effects on thyroid biosynthetic regulation that could have implications for inflammatory thyroid conditions, though direct evidence for immunomodulation in autoimmune contexts requires further investigation using appropriate autoimmune disease models.

The therapeutic efficacy of MEWS is attributed to its phytoconstituents, particularly withanolides, flavonoids, and phenolic compounds, which are enriched through methanolic extraction. These bioactives are known for their antioxidant, anti‐inflammatory, and immunomodulatory properties (Singirala et al. 2025; Vaidya et al. 2024). Our findings align with previous studies showing that hypothyroidism is associated with significantly elevated oxidative stress markers and reduced antioxidant enzymes (e.g., SOD, CAT, GPx, GSH) (A. Kar et al. 2022). The proposed antioxidant mechanism is supported by several lines of evidence: (i) phenolic compounds in *W. somnifera* , such as withanolides, are potent free radical scavengers that can directly neutralize reactive oxygen species (ROS); (ii) these compounds have been shown to upregulate nuclear factor erythroid 2‐related factor 2 (Nrf2) signaling, leading to increased expression of antioxidant response elements; and (iii) by reducing oxidative stress, MEWS may protect thyroid follicular cells from lipid peroxidation and membrane damage, thereby preserving TPO enzyme activity and hormone synthetic capacity (Alghamdi et al. 2024; Singirala et al. 2025). The reduction in thyroid hypertrophy and fibrosis observed in our histopathological analysis is consistent with this antioxidant‐mediated cytoprotection.

Beyond antioxidant effects, anti‐inflammatory mechanisms likely contribute to MEWS's therapeutic efficacy. Chronic inflammation, characterized by elevated pro‐inflammatory cytokines (IL‐1*β*, TNF‐*α*, IL‐6), can disrupt thyroid function by inhibiting TPO activity and promoting fibrosis (Vargas‐Uricoechea 2023). *W. somnifera* has been shown to suppress NF‐κB activation and reduce COX‐2 expression, thereby attenuating inflammatory responses in various tissues (Gupta and Singh 2014). The reduced vascular congestion and fibrosis observed in our MEWS‐treated groups may reflect such anti‐inflammatory activity, though direct measurements of cytokines in future studies would confirm this mechanism.

*In silico* docking provided predictive mechanistic insights into potential interactions between phytochemicals and target proteins. Oxitriptan showed promising predicted binding affinity to the TSH receptor (TSHR) and type 2 iodothyronine deiodinase (D2) in our computational models, with calculated affinities exceeding those of L‐thyroxine (see Table 1 for full docking scores). These *in silico* findings suggest a possible dual mechanism that warrants experimental investigation: the potential potentiation of TSH signaling via TSHR and possible enhancement of T4‐to‐T3 conversion via D2. However, it is important to emphasize that these computational predictions require validation through in vitro receptor‐binding assays and in vivo functional studies to confirm actual biological activity. Critically, the drug‐likeness and ADMET profile of these compounds are essential to evaluate their translational potential. All three phytochemicals satisfied Lipinski's Rule of Five, predicting good oral bioavailability. However, a deeper analysis reveals distinct advantages and challenges for each lead compound.

Based on our computational analyses, oxitriptan emerged with the most promising overall predicted profile. It demonstrated not only strong *in silico* binding affinity but also favorable predicted gastrointestinal absorption (55.59%), no predicted toxicity signals (Ames, hERG, hepatotoxicity), and no significant CYP450 inhibition liabilities *in silico* (Table 4). This computational ADMET profile, characterized by good predicted absorption and a clean safety prediction, suggests that oxitriptan warrants prioritization for further experimental validation through in vitro and in vivo studies.

In contrast, the other high‐binding compounds present specific predicted limitations that must be taken into account for future research prioritization. Caftaric acid, despite its strong docking scores, is predicted to have very low gastrointestinal absorption (9.40%) *in silico*, which could potentially limit its oral efficacy unless alternative formulations are developed. 6‐Benzylaminopurine, while showing excellent predicted absorption (93.26%), was flagged for potential toxicity risks in computational models, including mutagenicity (Ames test) and cardiotoxicity (hERG II inhibition). These computational predictions serve as early alerts that would require thorough experimental toxicological assessment.

These computational findings provide a plausible mechanistic framework that is consistent with our in vivo results, offering hypotheses for how MEWS phytochemicals might modulate thyroid hormone synthesis and signaling. Such hypotheses now require direct experimental testing.

Interestingly, the crude extract demonstrated greater efficacy than individual phytochemicals, underscoring the concept of phytochemical synergy. Whole‐extract preparations can leverage the additive and synergistic effects of multiple compounds, leading to enhanced antioxidant, anti‐inflammatory, and thyroid‐regulating actions. This is a recognized advantage of herbal extracts, where the combined effect of numerous bioactives often surpasses the efficacy of isolated molecules. For example, Williamson (2001) described “polyvalent synergy,” where multiple compounds in an extract act on different targets to produce a combined effect greater than the sum of individual activities (Williamson 2001). In the context of thyroid modulation, this synergy could operate through multiple complementary mechanisms: (i) some compounds (e.g., withaferin A) may provide antioxidant protection; (ii) others (e.g., oxitriptan) may interact directly with TSHR or D2; (iii) flavonoids may improve bioavailability of other constituents; and (iv) multiple compounds may together modulate inflammatory signaling cascades more effectively than any single agent. This concept of “network pharmacology” is increasingly recognized as a strength of traditional herbal preparations and supports the use of standardized *W. somnifera* extracts for thyroid therapy (Saleem et al. 2020; Tomer and Gupta 2025).

Nonetheless, several limitations should be acknowledged. First, while the PTU‐induced hypothyroidism model is well‐established for studying thyroid dysfunction, it primarily reflects chemically induced thyroid hormone deficiency and does not fully recapitulate the complex immune pathogenesis of autoimmune thyroiditis, such as Hashimoto's disease. Therefore, extrapolations to autoimmune contexts should be made with caution, and future studies using autoimmune models (e.g., spontaneous autoimmune thyroiditis in NOD.H‐2 h4 mice) are needed to evaluate MEWS's effects on thyroid autoantibody production and immune cell infiltration. Second, although our *in silico* docking provides valuable mechanistic predictions, experimental validation via receptor‐binding assays, enzyme kinetics, and pharmacokinetic studies is necessary. Third, future studies should explicitly measure oxidative stress markers (MDA, GSH), liver enzymes (ALT, AST), and inflammatory cytokines (TNF‐*α*, IL‐6) to quantitatively confirm the antioxidant and anti‐inflammatory mechanisms proposed. Moreover, autoimmune models and clinical settings should be explored to evaluate MEWS's impact on thyroid autoantibody production and its potential role in Hashimoto's thyroiditis. Future studies integrating transcriptomic and proteomic profiling will be essential to delineate its broader regulatory networks.

Taken together, this study establishes MEWS as a promising modulator of thyroid function, capable of restoring hormone balance, protecting thyroid morphology, and regulating key biosynthetic genes. Integration of in vivo and *in silico* evidence highlights oxitriptan as a lead phytochemical while emphasizing the synergistic efficacy of whole‐extract therapy. Future preclinical and clinical studies, including autoimmune models and randomized controlled trials, are warranted to validate safety, optimize dosing, and confirm translational potential. Collectively, these findings support *W. somnifera* as a complementary therapeutic candidate for hypothyroidism management.

## 5 Conclusion

This study demonstrates that the methanolic extract of *W. somnifera* root (MEWS) shows promise as a multi‐targeted therapeutic agent for hypothyroidism in a PTU‐induced rat model, with observed effects including restoration of hormone balance, protection of thyroid morphology, and regulation of key biosynthetic genes (TPO and TG). The integration of in vivo evidence with *in silico* predictions highlights oxitriptan as a lead phytochemical warranting further investigation and provides a mechanistic hypothesis for the observed efficacy, while also emphasizing the potential value of whole‐extract therapy. Future preclinical studies using autoimmune thyroiditis models, along with comprehensive toxicological assessments and randomized controlled trials, are necessary to validate safety, optimize dosing, and determine translational potential. Collectively, these findings suggest that *W. somnifera* merits further investigation as a complementary approach for hypothyroidism management, though additional research is required before clinical recommendations can be made.

## Author Contributions

**Md. Saud Hossain:** conceptualization (equal), methodology (equal), software (equal), validation (equal), writing – original draft (equal). **Md. Shah Poran Shuvo:** conceptualization (equal), methodology (equal), software (equal), validation (equal), writing – original draft (equal). **M. Maruf Hasan Talukder:** data curation (lead), software (lead), writing – review and editing (lead). **Shahidullah:** data curation (equal), writing – review and editing (equal). **Sumaia Jannat:** data curation (equal), writing – review and editing (equal). **S. M. Riaduzzaman Niaz:** data curation (equal), writing – review and editing (equal). **Md Sohel:** data curation (equal), writing – review and editing (equal). **Md. Khairul Islam:** supervision (lead), validation (lead), writing – review and editing (lead).

## Ethics Statement

This study was approved by the Ethical Review Committee of the Department of Biochemistry and Molecular Biology at Mawlana Bhashani Science and Technology University, Tangail‐1902, Bangladesh, with the certificate number MBSTU/BMB/TEST/2/2024/153.

## Consent

The authors have nothing to report.

## Conflicts of Interest

The authors declare no conflicts of interest.

## Supporting information


**Table S1:** Average thyroid gland weight (mg) among animal groups.
**Table S2:** TSH concentration (mIU/L).
**Table S3:** T3 concentration (ng/mL).
**Table S4:** T4 concentration (ng/mL).
**Table S5:** Ct value and RFC of TPO (thyroid peroxidase) gene.
**Table S6:** Ct value and RFC of TG (thyroglobulin) gene.

## Data Availability

The information backing up this study's results can be obtained from the lead author by making a reasonable request. Employed web servers include phytochemicals extracted from IMPAAT (https://cb.imsc.res.in/imppat/), PubChem (https://pubchem.ncbi.nlm.gov/), 3D structure of protein from RCSB Protein Data Bank (https://www.rcsb.org), ADMET properties from SwissADME (http://www.swissadme.ch.), and pkCSM (https://biosig.lab.uq.edu.au/pkcsm/prediction).
