# Supplementary material for: Withania somnifera Root Extract Ameliorates PTU‐Induced Hypothyroidism by Regulating Hormone Levels and Gene Expression in Rats
Source: Food Sci Nutr. 2026 Apr 17;14(4):e71732. doi: 10.1002/fsn3.71732 (PMC13088288; doi:10.1002/fsn3.71732)
Supplement: Supplementary file 1 — Table S1: Average thyroid gland weight (mg) among animal groups. Table S2: TSH concentration (mIU/L). Table S3: T3 concentration (ng/mL). Table S4: T4 concentration (ng/mL). Table S5: Ct value and RFC of TPO (thyroid peroxidase) gene. Table S6: Ct value and RFC of TG (thyroglobulin) gene. [file FSN3-14-e71732-s001.docx]

**Supplementary Table 1: Average Thyroid Gland Weight (mg) among Animal Groups**

| **Animal Group** | **Average Thyroid Gland Weight (mg)** |
| --- | --- |
| **NC  Negative Control (Healthy)** | 36 |
| **WSC *Withania somnifera* Control** | 35.6 |
| **PC Positive Control (Diseased)** | 99.3 |
| **WST-500  *Withania somnifera* Treatment 500 mg/kg/day** | 44 |
| **CT-500  Combined therapy (MEWS + PTU)** | 50.3 |

**Note****:** Average thyroid gland weight (mg) in different experimental animal groups.

**Supplementary Table 2: TSH Concentration (mIU/l)**

| **Animal**  **Group** | **Average TSH Concentration (mIU/l)** | **Standard Deviation & Standard Error** |
| --- | --- | --- |
| **NC  Negative Control (Healthy)** | 4.29 | SD=1.46 & SE=0.6 |
| **WSC *Withania somnifera* Control** | 4.5 | SD=1.77 & SE=0.72 |
| **PC Positive Control (Diseased)** | 19.08 | SD=2.7 & SE=1.1 |
| **WST-500  *Withania somnifera* Treatment 500 mg/kg/day** | 4.92 | SD=2.04 & SE=0.83 |
| **Group-5  Synergistic Effects after Diseased** | 6.58 | SD=2.19 & SE=0.89 |

**Note:** Serum TSH concentration (mIU/L) with corresponding standard deviation (SD) and standard error (SE) across animal groups.

**Supplementary Table 3: T3 Concentration (ng/ml)**

| **Animal**  **Group** | **Average T3 Concentration (ng/ml)** | **Standard Deviation & Standard Error** |
| --- | --- | --- |
| **NC  Negative Control (Healthy)** | 7.00 | SD=1.26 & SE=0.51 |
| **WSC *Withania somnifera* Control** | 7.00 | SD=1.26 & SE=0.51 |
| **PC Positive Control (Diseased)** | 1.26 | SD=0.92 & SE=0.38 |
| **WST-500  *Withania somnifera* Treatment 500 mg/kg/day** | 6.72 | SD=1.24 & SE=0.51 |
| **CT-500  Combined therapy (MEWS + PTU)** | 5.24 | SD=1.78 & SE=0.73 |

**Note:** Serum T3 concentration (ng/mL) with SD and SE across animal groups.

**Supplementary Table 4: T4 Concentration (ng/ml)**

| **Animal**  **Group** | **Average T4 Concentration (ng/ml)** | **Standard Deviation & Standard Error** |
| --- | --- | --- |
| **NC  Negative Control (Healthy)** | 2.00 | SD=0.36 & SE=0.15 |
| **WSC *Withania somnifera* Control** | 2.00 | SD=0.36 & SE=0.15 |
| **PC Positive Control (Diseased)** | 0.50 | SD=0.23 & SE=0.09 |
| **WST-500  *Withania somnifera* Treatment 500 mg/kg/day** | 1.80 | SD=0.34 & SE=0.14 |
| **CT-500  Combined therapy (MEWS + PTU)** | 1.50 | SD=0.43 & SE=0.18 |

**Note:** Serum T4 concentration (ng/mL) with SD and SE across animal groups

**Supplementary Table 5: Ct Value and RFC of TPO (Thyroid Peroxidase) Gene**

| **Animal**  **Group** | **Ct value** | **SD&SE** | **RFC** | **SD&SE** |
| --- | --- | --- | --- | --- |
| **NC  Negative Control (Healthy)** | 33.88 | SD=0.56  SE=0.23 | 1 | SD=0.67  SE=0.27 |
| **WSC Withania somnifera Control** | 33.76 | SD=0.83  SE=0.34 | 1.03 | SD=0.75  SE=0.31 |
| **PC Positive Control (Diseased)** | 31.66 | SD=1.18  SE=0.45 | 4.28 | SD=0.85  SE=0.32 |
| **WST-500  Withania somnifera Treatment 500 mg/kg/day** | 33.29 | SD=0.95  SE=0.39 | 1.35 | SD=0.72  SE=0.29 |
| **CT-500  Combined therapy (MEWS + PTU)** | 32.63 | SD=1.01  SE=0.41 | 1.85 | SD=0.94  SE=0.38 |

**Note:** Ct values and relative fold change (RFC) of thyroid peroxidase (TPO) gene expression in experimental groups, with SD and SE.

**Supplementary Table 6: Ct Value and RFC of TG (Thyroglobulin) Gene**

| **Animal**  **Group** | **Ct value** | **SE** | **RFC** | **SE** |
| --- | --- | --- | --- | --- |
| **NC  Negative Control (Healthy)** | 34.94 | 0.46 | 1 | 0.55 |
| **WSC Withania somnifera Control** | 34.83 | 0.49 | 0.99 | 0.55 |
| **PC Positive Control (Diseased)** | 32.77 | 0.40 | 3.10 | 0.60 |
| **WST-500  Withania somnifera Treatment 500 mg/kg/day** | 34.50 | 0.48 | 1.30 | 0.69 |
| **CT-500  Combined therapy (MEWS + PTU)** | 33.69 | 0.30 | 1.36 | 0.31 |

**Note:** Ct values and RFC of thyroglobulin (TG) gene expression in experimental groups, with SE.
